# A 46-Year-Old Presenting With Inferior Mononuclear Visual Field Defect as the Sole Manifestation of Neurosarcoidosis

**DOI:** 10.7759/cureus.13076

**Published:** 2021-02-02

**Authors:** Carl Hoegerl, Addie Amper

**Affiliations:** 1 Internal Medicine and Neurology, Centra Health and Liberty University College of Osteopathic Medicine, Lynchburg, USA; 2 Neurology, Liberty University College of Osteopathic Medicine, Lynchburg, USA

**Keywords:** neurosarcoidoisis, sarcoidosis, quadrantanopsia, vision loss

## Abstract

Neurosarcoidosis (NS) is a rare manifestation of sarcoidosis that lacks an organized body of knowledge regarding its diagnosis and management. There exists no clearly defined diagnostic criteria to make the diagnosis. To further complicate things, biopsy of the nervous system tissue remains complicated and not easily accomplished due to the sensitivity of the tissue and the subsequent deficits it could produce. In this case report, we present a patient who presented with acute inferior mononuclear quadrantanopsia with an insignificant past medical history and a lack of other signs and symptoms. Follow-up studies and magnetic resonance imaging of the orbits eventually led to the diagnosis of NS.

## Introduction

Sarcoidosis is a granulomatous disease whose cause remains unknown [1]. It can affect multiple organs in the body, including the lungs, heart, and nervous system. When it affects the nervous system, it is referred to as neurosarcoidosis (NS). It can cause very severe deficits in a patient if the nervous system is involved. Its ability to affect any organ system in the body shows how complex NS is and further necessitates how research about its causes and complications is extremely important. Both the etiology and pathophysiology of NS are still unclear. NS is often dismissed due to its rarity and most think of the pulmonary manifestations of the disease.

Because of its potential severity and the lack of literature surrounding its diagnosis and management, NS is a worthy topic of discussion. Currently, there is a reported collection of symptoms that have been noted as NS. These symptoms range from blurry vision and floaters to a skin tattoo reaction [2].

## Case presentation

A 46-year-old male presented with sudden visual disturbance that started one month prior to presentation. The patient had been having problems with blurred vision, loss of peripheral vision, and floaters in his left eye only. He was specific in that his symptoms were only occurring out of his left eye. He stated that his symptoms started with his central vision loss and then he could not see out of the peripheral part of his vision. He did have a history of ocular migraines, but these symptoms were different than his usual migraines. These new symptoms were persistent and not intermittent. In addition, he had just had a full eye examination by a neuro-ophthalmologist the week before and no eye abnormalities were found. The patient denied any events that provoked or alleviated the complaint. There was no significant medical and social history related to his chief complaint. He worked as a construction worker and reported a significant amount of stress in his life. Magnetic resonance imaging (MRI) of the brain and carotid ultrasound were recently done and were reported as normal.

Past medical history included a history of lumbar back pain, gastroesophageal reflux, allergic rhinitis, and depression. He had a minor surgery as a child for his knees and a broken ankle. He was married, non-smoker, and worked full time as a construction worker. Family history was otherwise unremarkable. He had no recent foreign travel history. Review of systems was otherwise normal.

On physical examination, his vitals were normal. He was awake, alert, and oriented, and his mental status was normal. He had a normal eye examination except that he had a monocular left-sided inferior visual field defect (confirmed by formal visual field testing). Right eye examination was normal including visual eye fields. The remainder of the neurological and physical examination was normal. 

**Figure 1:**
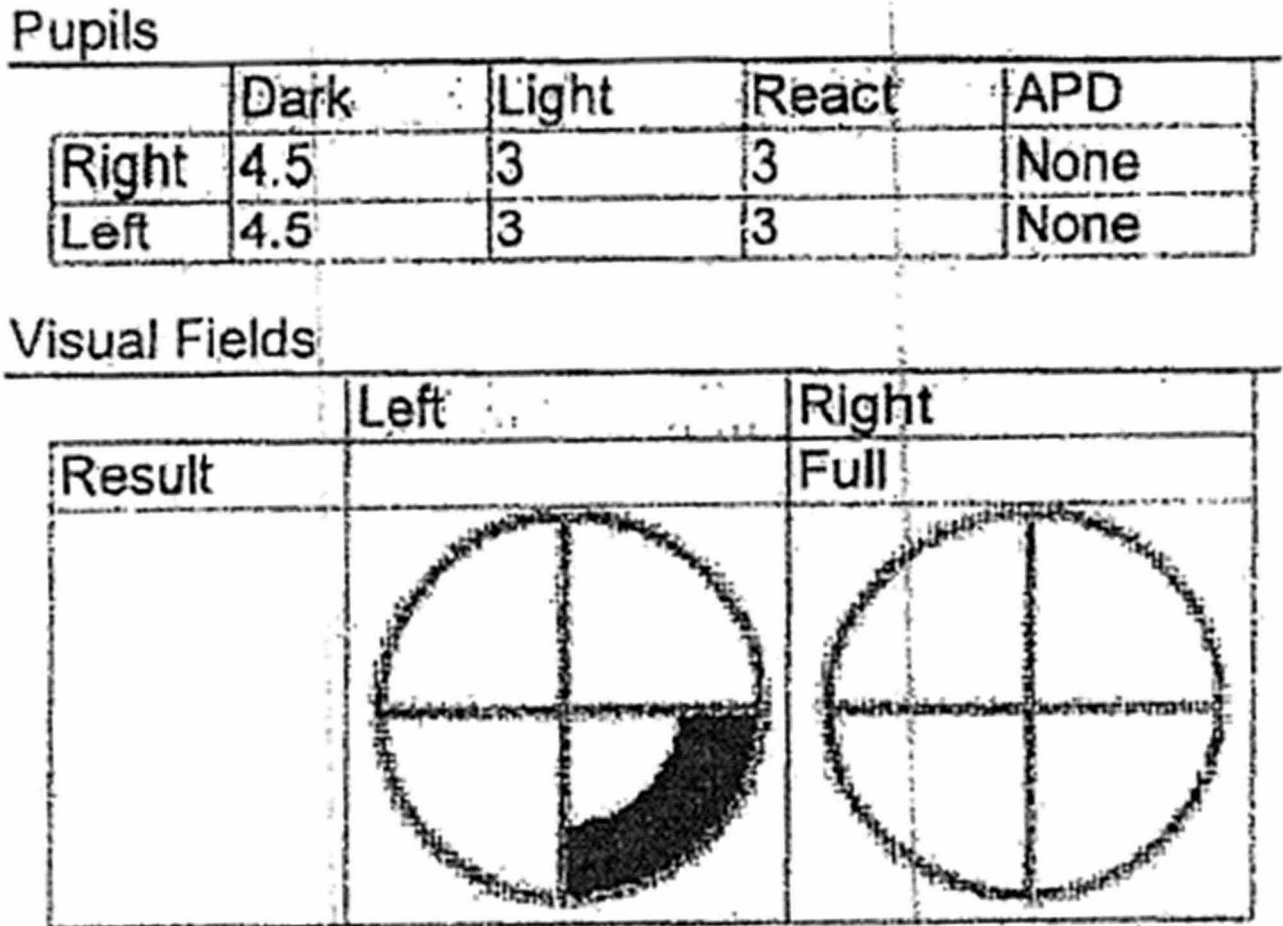
Visual field mapping showing a left inferior quadrant nasal visual defect.

Initially, as the eye examination was normal and the symptoms were monocular, an assumption was made that the symptoms were from stress and that, with time, the symptoms would resolve.

On a subsequent visit one week later, the patient returned for follow-up. Despite taking some time off from work and reducing the stress level in his life, the patient still had persistent symptoms of left-sided monocular inferior quadrantanopsia. Upon insistence from the patient, a decision was made to repeat MRI of the optic nerves. In addition, the patient proceeded to tell the team that he had neglected to tell us that he was worked up for sarcoidosis a few years previously but no definitive conclusion had ever been made.

Follow-up imaging of the optic nerves showed a small contrast-enhancing lesion on the left optic nerve. It was suspected that the patient may have sarcoidosis. He was also beginning to have some modest breathing difficulties.

**Figure 2:**
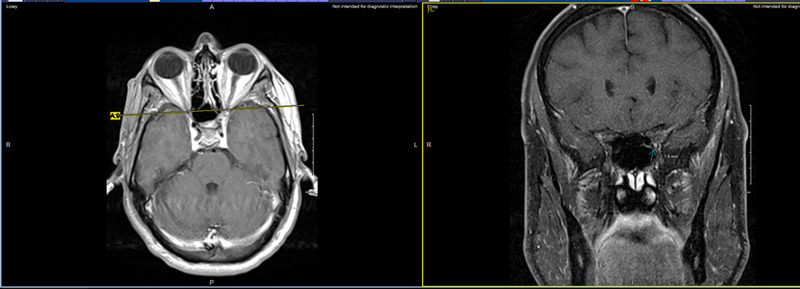
Small enhancing lesion in the left optic nerve indicated by the arrow. Both the axial and coronal views can be seen.

Subsequent assessment by bronchoscopy with biopsy confirmed a diagnosis of sarcoidosis. Upon further review of his MRI with neuro-radiology, a determination was made that the patient likely had NS causing the symptoms of his left eye. The patient responded well to treatment and the symptoms resolved.

In addition, further evaluation by both pulmonology and rheumatology helped to rule out other causes of granulomatous disease.

## Discussion

NS presents in 5% of patients who have sarcoidosis [3]. In addition, establishing a diagnosis of NS has been difficult because until recently there were no established criteria and getting a nerve tissue biopsy was tough [4].

However, in 2018, the Neuro-sarcoidosis Consortium Consensus Group established some baseline criteria that clinicians can use to help establish a diagnosis of NS [5]. Furthermore, in 2019, international criteria for the diagnosis of ocular sarcoidosis was updated and more clearly delineated [6]. Based on this criteria, the patient described in this report met the criteria for NS. Establishing the diagnosis based on these criteria makes the diagnosis and treatment much easier than before. Trying to establish the diagnosis of NS with tissue biopsy exclusively is dangerous because of the sensitivity of any neural tissue that needs to be taken. In this case, tissue biopsy was successful with follow-up bronchoscopy and tissue confirmation.

In the case presented here, the patient presented with monocular vision loss that was in the lower quadrant (nasal) of vision in one eye only. Most of the time, this type of vision loss presents in a patient in a binocular presentation. This presentation is also highly unusual to begin with. What makes this case even more unique is that this vision loss was associated with NS.

The causes of binocular and monocular vision loss are really different. Binocular vision loss is due to a lesion that occurs in optic nerve posterior to the optic chiasm and often involves a lesion in the brain such as a stroke. Monocular vision loss involves either the eye itself or a lesion that is between the eye and the optic chiasm. Table 1 lists some of the potential causes of monocular vision loss.

Overall, this case adds a great deal to the research literature in that few cases of ocular sarcoidosis present in this unique manner.

**Table 1:** Differential diagnosis for monocular vision loss.

Causes of monocular vision loss
Glaucoma
Amaurosis fugax (ischemia)
Orbital tumor
Retinal artery occlusion
Papilledema
Optic neuritis
Trauma
Migraine

## Conclusions

Once a diagnosis of NS is made, proper surveillance and treatment can be initiated. Although there is still a long way to go in understanding NS, significant treatment progress has been made in recent years and will likely continue. As with many things in medicine, progress will help us understand NS better so that treatment can be improved. Hopefully, one day, a cure will be found for this.

Usually, when a patient presents with complaints of vision loss in one eye only, the differential usually falls on the eye itself and what could be causing vision loss in the eye. When there is a bilateral presentation of vision loss, a lesion in the brain is suspected (i.e., stroke). In this case, the lesion was between the eye and the optic chiasm. This explains why the eye examination itself (except visual fields) was normal and the vision loss was monocular in nature.

Another lesson and great clinical take-home point or pearl from this article is that NS can present in various ways. There is not only one way that NS can present. In this case, where there is a unilateral visual presentation of NS, it can easily confuse the physician. It is easy for the physician to dismiss the patient and not take the symptoms seriously. In this case, in particular, that could have been devastating for the patient.
